# Supplementary material for: High-dimensional normalized data profiles for testing derivative-free optimization algorithms
Source: PeerJ Comput Sci. 2022 Jul 22;8:e960. doi: 10.7717/peerj-cs.960 (PMC9454945; doi:10.7717/peerj-cs.960)
Supplement: Supplemental Information 1 — The function set includes a variety of optimization problems. The scripts show how the proposed algorithm can tackle those problems efficiently. A reviewer can easily run and debug the code. [file peerj-cs-08-960-s001.gz › C# scripts/Read me.docx]

Read me:

The supplementary materials are programs written in C# language, which provide a prospective on how the proposed algorithm, Multidirectional Trigonometric Nelder Mead algorithm, (MTNMa) manages to find an optimum. I tried to demonstrate MTNMa through a mathematical model and a full description in the article. I found it is necessary to have the programs appended to the article. Since some of the parameters are used in the article from programming point of view. If someone has a consolidate full of understanding to my article, it is still some points need to be clarified. And I would be very happy to work or cooperate with anyone wants to take the high dimensional data profiles to the next level.

It is preferable to use C# software to run and debug the programs. Once a script is launched, you need to provide number of simplexes. You can choose the same number of simplexes provided in Table 1 in the manuscript.
